# Supplementary material for: Dissociable effects of dopaminergic medications on depression symptom dimensions in Parkinson disease
Source: Nat Ment Health. 2024 Jun 17;2(8):916–23. doi: 10.1038/s44220-024-00256-8 (PMC11310074; doi:10.1038/s44220-024-00256-8)
Supplement: Supplementary file 1 — Supplementary Information Supplementary Figs. 1–3, Q–Q plots and Tables 1 and 2. [file 44220_2024_256_MOESM1_ESM.pdf]

nature mental health

Supplementary information

https://doi.org/10.1038/s44220-024-00256-8

Dissociable effects of dopaminergic medications on depression symptom dimensions in Parkinson disease

In the format provided by the authors and unedited

Supplement QQ plots: residuals of the model are plotted against the quantiles of a standard normal distribution. When the normality assumption is met, the residuals will align with the quantiles of the standard normal distribution, resulting in a relatively straight diagonal line.

1. Linear motivation factor model –

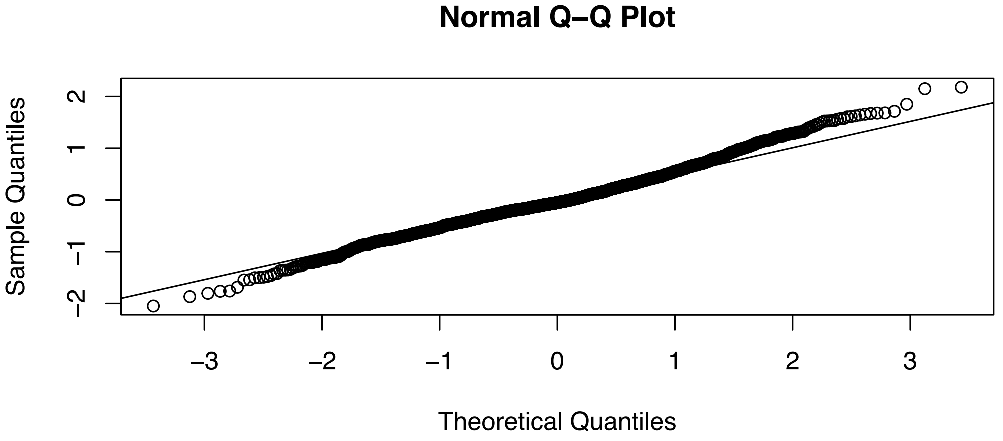

2. Linear depression factor model –

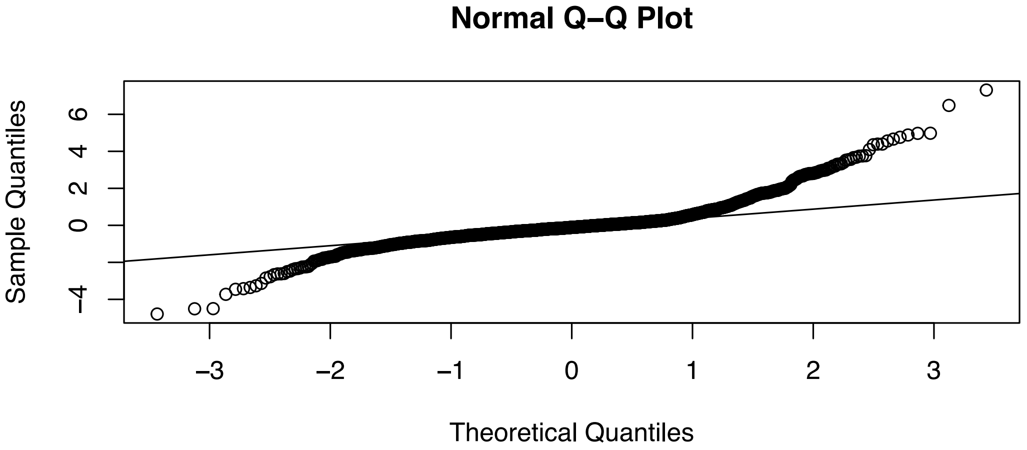

3. Linear depression factor model following logarithmic transformation –

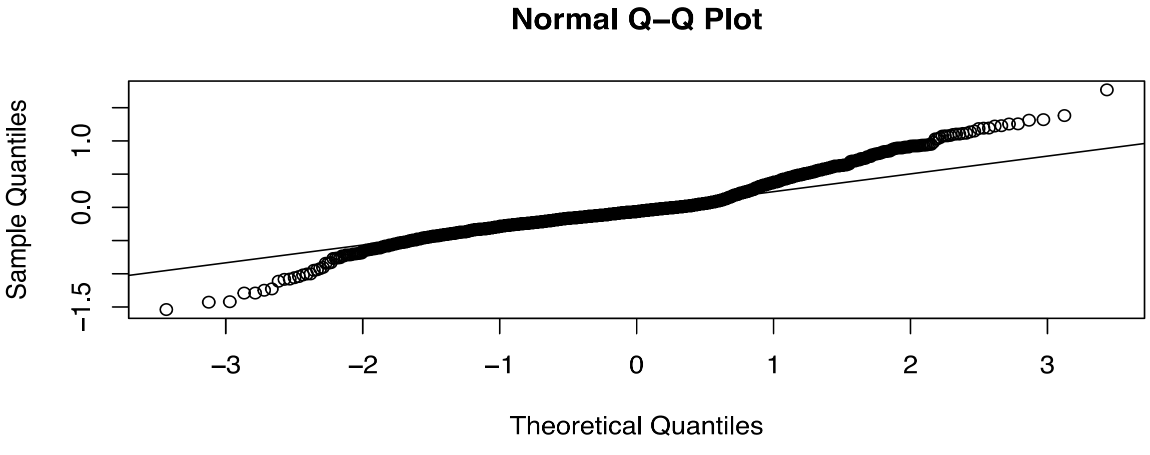

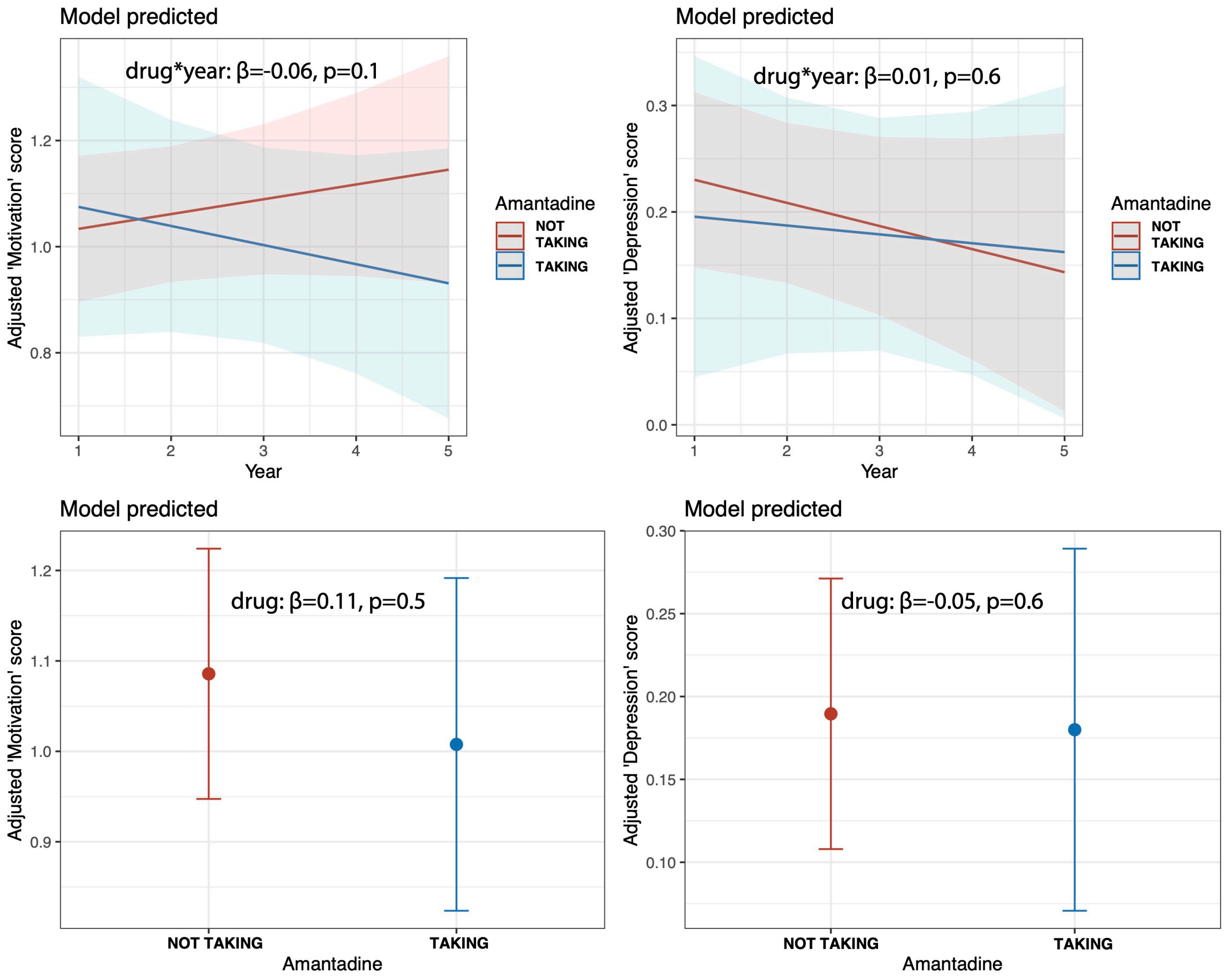

Supplement figure S1. Top row. Adjusted linear mixed effects model of predicted relationship between amantadine treatment and 'motivation' factor score (top left) and 'depression' factor score (top right) over time (shown as the mean estimate (red & blue lines) ±95% confidence intervals (red & blue shading).

Bottom row. Adjusted linear mixed effects model predicted relationship between amantadine treatment and 'motivation' factor score (bottom left) and 'depression' factor (bottom right) score across all timepoints (shown as mean estimate +/- two standard errors).

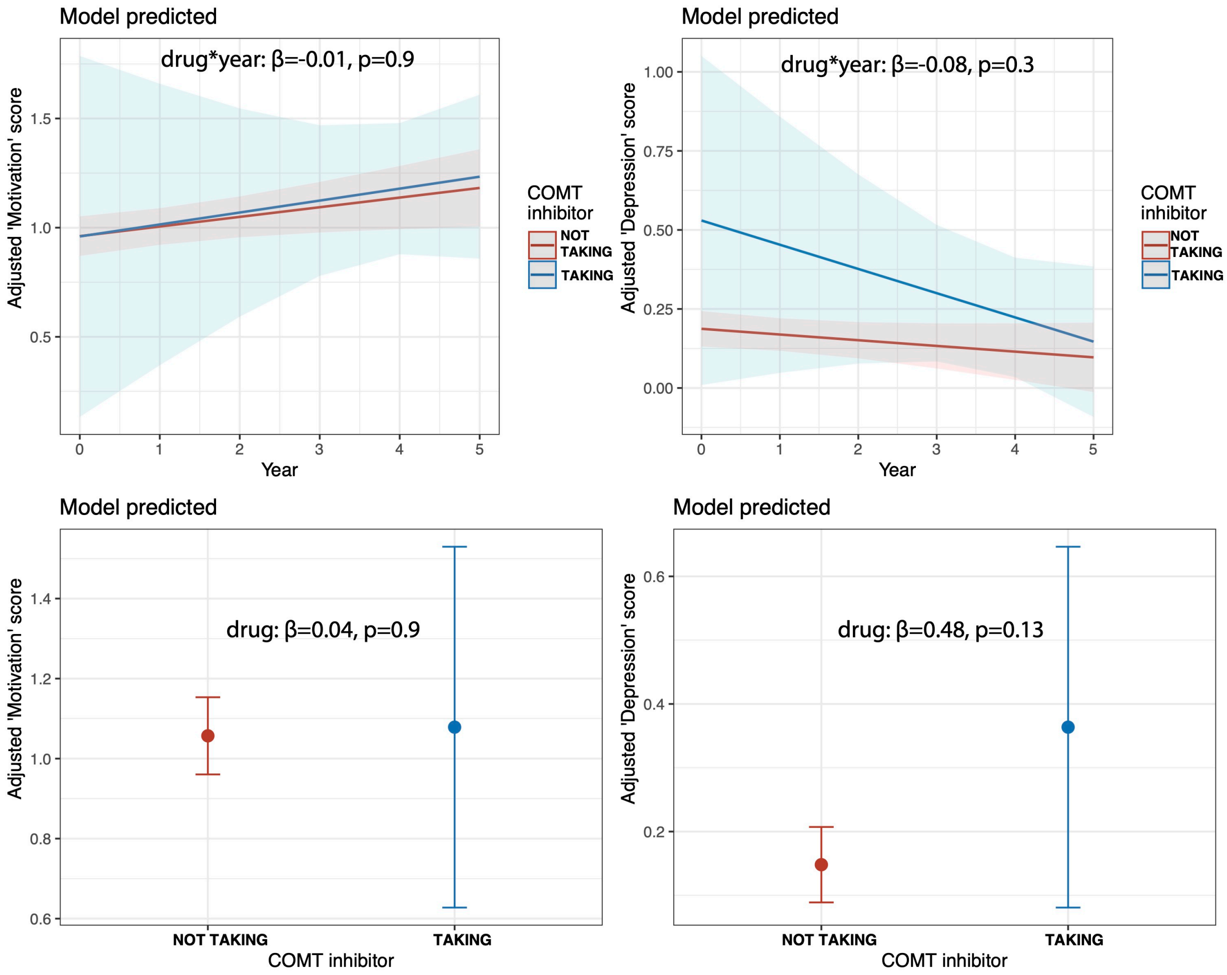

Supplement figure S2. Top row. Adjusted linear mixed effects model predicted relationship between COMT inhibitor treatment and ‘motivation’ factor score (top left) and ‘depression’ factor score (top right) over time (shown as the mean estimate (red & blue lines) ±95% confidence intervals (red & blue shading). **Bottom row.** Adjusted linear mixed effects model predicted relationship between COMT inhibitor treatment and ‘motivation’ factor score (bottom left) and ‘depression’ factor score (bottom right) across all timepoints (shown as mean estimate +/- two standard errors).

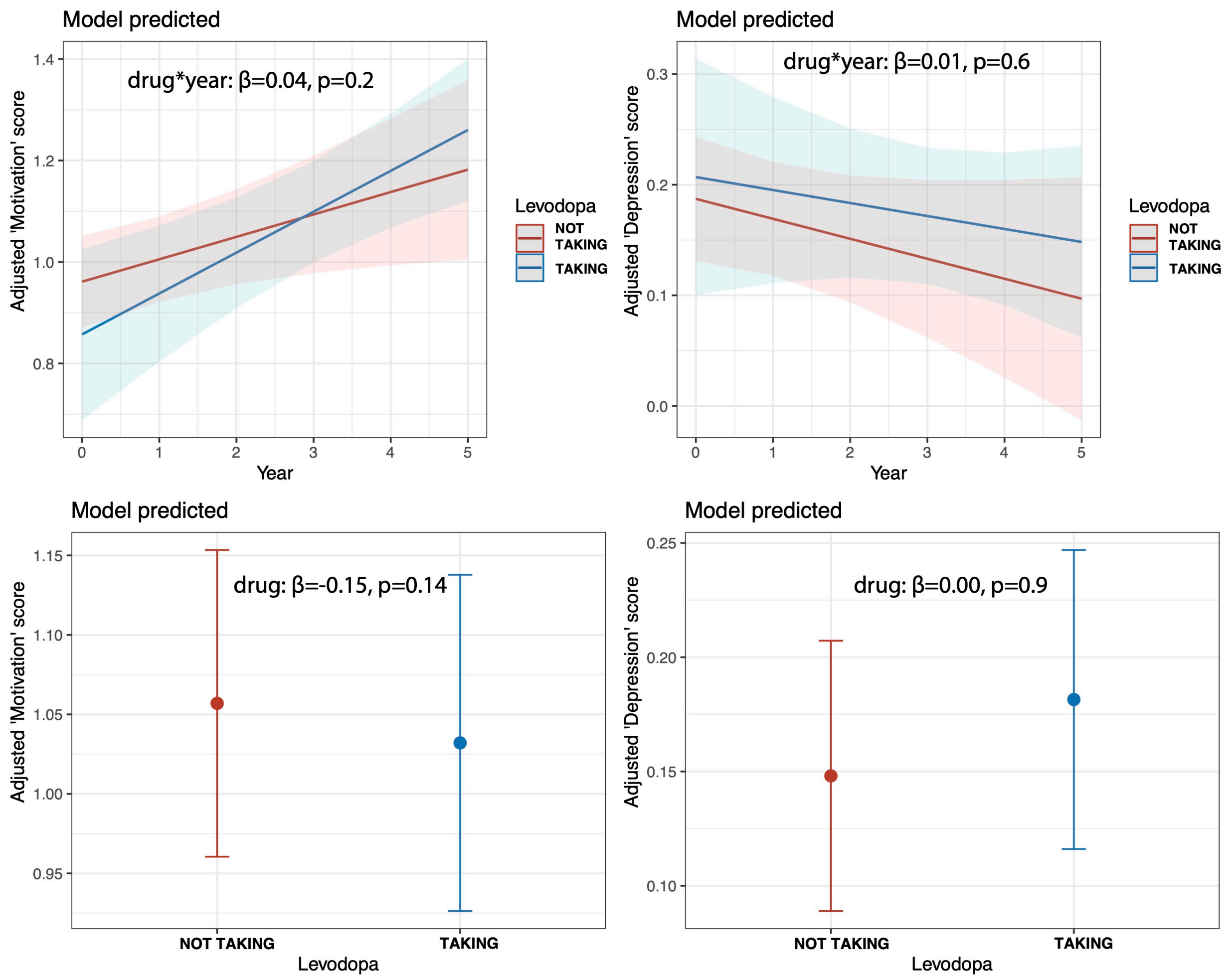

Supplement figure S3. Top row. Adjusted linear mixed effects model of predicted relationship between levodopa treatment and 'motivation' factor score (top left) and 'depression' factor score (top right) over time (shown as the mean estimate (red & blue lines) ±95% confidence intervals (red & blue shading).

Bottom row. Adjusted linear mixed effects model predicted relationship between levodopa treatment and 'motivation' factor score (bottom left) and 'depression' factor score (bottom right) across all timepoints (shown as mean estimate +/- two standard errors).

Supplement table 1. Adjusted mixed-effects model results investigating the relationship between PD medication and odds of developing clinically significant apathy/anhedonia (motivation score ≥2) longitudinally

**Medication**	**Odds ratio (OR) of high apathy/anhedonia ((motivation score ≥2)**	
**Dopamine agonists**	**drug*time**	**OR=0.68, 95%CI [0.49, 0.94], p=0.021***
**Levodopa**	**drug*time**	OR=1.18, 95%CI[0.85, 1.65]
**MAO-B inhibitors**	**drug*time**	OR=0.76, 95%CI[0.55, 1.03]
**COMT inhibitors**	**drug*time**	OR=0.57, 95%CI[0.12, 2.65]
**Amantadine**	**drug*time**	OR=0.79, 95%CI[0.52, 1.19]
* p<0.05 ** p<0.01, statistically significant results in bold.		

Supplement table 2. Adjusted mixed-effects model results investigating the relationship between PD medication and odds of developing clinically significant apathy/anhedonia (motivation score ≥2) by striatal dopamine transporter (DAT) specific binding ratio (SBR)

**Medication**	**Odds ratio (OR) of high apathy/anhedonia ((motivation score ≥2)**	
**Dopamine agonists**	**drug*DAT SBR**	OR=1.68, 95%CI [0.66, 4.28]
**Levodopa**	**drug*DAT SBR**	OR=0.58, 95%CI [0.28, 1.21]
**MAO-B inhibitors**	**drug*DAT SBR**	**OR=0.28, 95%CI [0.11, 0.73], p=0.009****
**COMT inhibitors**	**drug*DAT SBR**	OR=302, 95%CI [0.25, 367]
**Amantadine**	**drug*DAT SBR**	OR=3.30, 95%CI [0.92, 11.85]
* p<0.05 ** p<0.01, statistically significant results in bold.		
